# State of Health Estimation and Battery Management: A Review of Health Indicators, Models and Machine Learning

**DOI:** 10.3390/ma18010145

**Published:** 2025-01-02

**Authors:** Mei Li, Wenting Xu, Shiwen Zhang, Lina Liu, Arif Hussain, Enlai Hu, Jing Zhang, Zhiyu Mao, Zhongwei Chen

**Affiliations:** 1 College of Chemistry and Materials Science, Zhejiang Normal University, 688 Yingbin Avenue, Jinhua 321004, China; liwangfanwen@zjnu.edu.cn (M.L.); leonxu@zjnu.edu.cn (W.X.); 599232641@zjnu.edu.cn (S.Z.); linaliu@zjnu.edu.cn (L.L.); arifhussain@zjnu.edu.cn (A.H.); huenlai@zjnu.edu.cn (E.H.); jingzhang@zjnu.edu.cn (J.Z.); 2 Power Battery & System Research Center, Dalian Institute of Chemical Physics, Chinese Academy of Sciences, Dalian 116023, China; 3 State Key Laboratory of Catalysis, Dalian Institute of Chemical Physics, Chinese Academy of Sciences, Dalian 116023, China

**Keywords:** lithium-ion batteries, estimated state-of-health, extracted health indicator, model, machine learning

## Abstract

Lithium-ion batteries are a key technology for addressing energy shortages and environmental pollution. Assessing their health is crucial for extending battery life. When estimating health status, it is often necessary to select a representative characteristic quantity known as a health indicator. Most current research focuses on health indicators associated with decreased capacity and increased internal resistance. However, due to the complex degradation mechanisms of lithium-ion batteries, the relationship between these mechanisms and health indicators has not been fully explored. This paper reviews a large number of literature sources. We discuss the application scenarios of different health factors, providing a reference for selecting appropriate health factors for state estimation. Additionally, the paper offers a brief overview of the models and machine learning algorithms used for health state estimation. We also delve into the application of health indicators in the health status assessment of battery management systems and emphasize the importance of integrating health factors with big data platforms for battery status analysis. Furthermore, the paper outlines the prospects for future development in this field.

## 1. Introduction

The creation and application of new renewable energy sources have gained significant attention as a solution to global energy challenges and environmental degradation, particularly due to their rapid development. High-efficiency energy storage technologies are essential for advancing sustainable energy solutions. Among these technologies, lithium-ion batteries have garnered considerable interest due to their high energy density, low self-discharge rate, long lifespan, and relatively lower environmental impact [1,2,3]. They are widely utilized in electric vehicles, electronic devices, military applications, and various other industries [4].

However, it is important to acknowledge that the operational lifespan of lithium-ion batteries tends to degrade over time. The mechanisms behind this degradation are complex and arise from several interrelated factors, including changes at the electrode/electrolyte interface; loss of active materials; and alterations in current collectors, conductive additives, and porosity [5]. Moreover, the aging mechanisms can vary depending on different operating conditions and involve insights from multiple interdisciplinary fields [6]. This complexity makes monitoring and predicting the battery’s state of health (SOH) crucial for understanding and managing the degradation process, which, in turn, facilitates better control over the battery’s performance and overall lifespan.

In extensive research, SOH is typically defined as the ratio of the actual capacity to the rated capacity of the battery. An accurate estimation of SOH enables predictions about the remaining service life of lithium-ion batteries, ultimately extending their overall lifespan. Energy storage is also pivotal in supporting smart grid and renewable energy systems [7,8]. However, over time, the performance of energy storage systems diminishes, leading to reduced charging and discharging capacity and economic benefits. Therefore, estimating the SOH of batteries within these systems is essential [9].

To improve SOH estimation, researchers have focused on extracting health indicators (HIs). For instance, Yue et al. [10] proposed a method for estimating and predicting SOH based on the information entropy of characteristic data, successfully achieving short-term SOH estimation for energy storage power stations using a neural network. Similarly, Chang et al. [11] examined the long-term SOH of lithium-ion battery packs in energy storage and developed a regression model that considers critical factors such as capacity, resistance, and state of charge (SOC). This model not only reflects the aging of battery cells but also addresses utilization concerns arising from consistent battery deterioration. Thus, the extraction of health indicators plays a key role in optimizing the performance of energy storage systems, enhancing energy utilization, and minimizing waste. By closely monitoring SOH, it is possible to detect aging trends in batteries in real time, thereby avoiding potential failures that could lead to system malfunctions. Moreover, understanding HIs can help formulate suitable charge and discharge strategies that extend battery service life [12].

In the realm of transportation, the development of electric vehicles represents an accepted clean technology that also addresses excessive carbon emissions [13]. Within electric vehicles, estimating the SOH of batteries through HIs is instrumental in optimizing driving performance and extending the vehicle’s range. As shown in Table 1, various online estimation methods are employed for early warning systems, necessitating that these methods perform effectively in real-world applications and can be integrated into actual battery management systems (BMSs) to enhance performance [14,15,16]. For instance, Wu et al. [17] utilized HIs extracted from charging capacity and incremental capacity to estimate SOH in lithium-ion batteries within electric vehicles, employing a ridge regression model to achieve more reliable predictions. Moreover, several data-driven AI approaches have gained traction in estimating SOH for lithium-ion batteries in automotive applications [18].

Despite the advancements in SOH estimation techniques, current research predominantly revolves around algorithm innovation, often overlooking systematic explorations of the health indicators employed in state of health estimation. As shown in Table 1, various reviews have contributed to the discourse on SOH estimation; however, gaps remain, particularly in the understanding of how HIs correlate with the underlying aging mechanisms of lithium-ion batteries. Given the intricate nature of lithium-ion battery degradation, there is an urgent need for further investigation into the relationship between extracted HIs and associated aging processes. This paper aims to fill that gap by summarizing existing SOH estimation methods and, through an extensive review of the relevant literature on HIs, elucidating the connections between HIs and aging mechanisms. Additionally, we will explore the decay mechanisms of batteries, offering valuable insights for enhancing battery performance and expanding their applications in the future.

**Figure 1:**
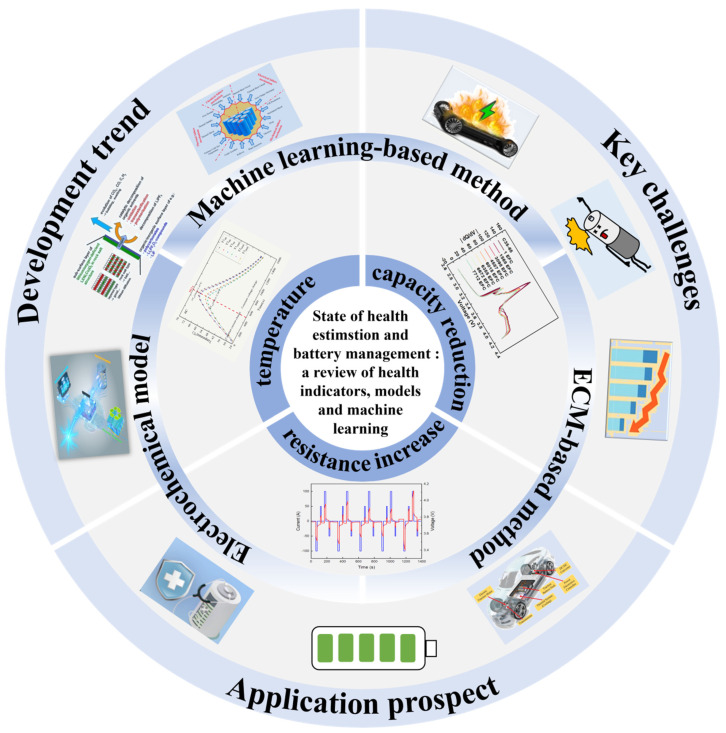
The main framework of the review. The subgraphs are from [25,26,27,28,29,30,31,32]; all drawings not otherwise noted are by the author.

This paper is organized as follows: Section 1 introduces the importance of lithium-ion batteries and their applications; Section 2 categorizes and defines health factors and evaluates methods and techniques for different health indicators. Section 3 summarizes estimation models for health indicators, while Section 4 discusses the development trends in health state estimation applications utilizing these indicators. Finally, Section 5 presents conclusions, addressing current challenges and future application prospects. The framework of this article is shown in Figure 1.

## 2. Definition and Classification of Health Indicators

### 2.1. Definition

**Figure 2:**
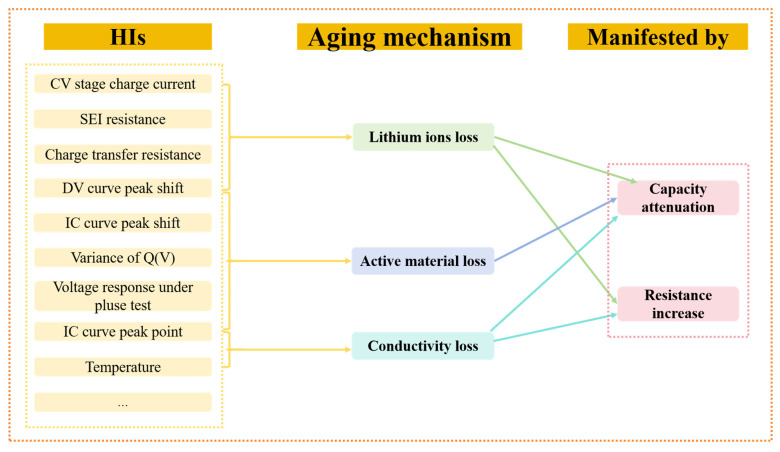
Relationships between HIs and aging mechanisms.

The battery reaches its end-of-life point when the usable capacity decreases to 80% of the standard capacity during use [33]. HI is an indicator used to evaluate the battery’s health status. Through extensive data mining, various data on the battery can be extracted and analyzed to calculate the health factor of the battery. The data and indicators that can be used to extract HI to characterize the battery status include battery capacity, charging speed, temperature data, number of cycles, etc. The relationship between some of the extracted HIs and the aging mechanism of the battery is summarized in Figure 2. Through mining and analyzing these data indicators, a model of battery health status can be established, and the HI value can be calculated, thus achieving accurate assessment and monitoring of the battery status. This helps to detect battery problems in advance, extend battery life, and improve the performance and reliability of the device. Therefore, the mechanism of battery aging is classified by identifying and quantifying HI and its changes during aging.

### 2.2. Classification of Health Indicators

#### 2.2.1. Correlated with Capacity Decrease

**Figure 3:**
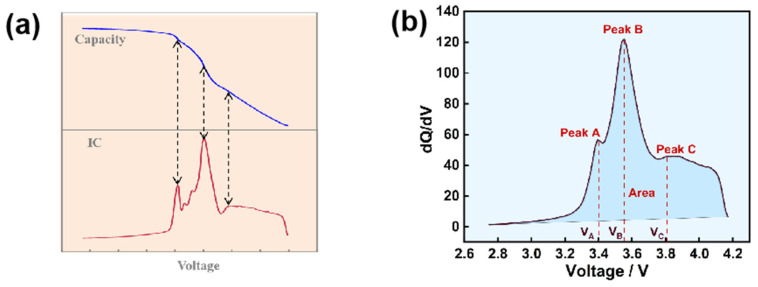
(**a**) Differential processing method in IC curve. (**b**) Extractable features in IC curves.

During the use of a battery, the capacity will decrease with the increase in the number of cycles. Hence, the capacity is a representative feature for estimating the battery’s health. The extraction of capacity-related HI is also significant work; according to a large number of studies, the characteristic quantity extracted from the incremental capacity analysis (ICA) curve and differential voltage analysis (DVA) curve has a strong correlation with the battery capacity decay of the dQ/dV and dV/dQ curves. As shown in Figure 3a, the incremental capacity (IC) curve of nickel–cobalt–manganese (NCM) ternary battery can be taken as an example using the differential idea of “straight” to “peak” and “slope” to “peak”. Converting a platform in the charge and discharge curve into an easily observable peak can achieve in situ nondestructive analysis of LIB, and monitoring battery aging to avoid complex post mortem analysis [34], which is especially suitable for exploring the capacity attenuation mechanism of storage and circulation processes combined with the differential curve of three electrodes, can solve the problem of peak identification and help to distinguish the active material loss and lithium ion loss. Therefore, exploring the relationship between the IC curve, differential voltage (DV) curve, and the aging mechanism is of great significance for accurately estimating the SOH of lithium-ion batteries.

The expression of ICA is shown in Equation (1), where *Q* represents the capacity of the battery and *V* represents the voltage of the battery:

(1) $$\frac{dQ}{dV}=\frac{Q_{2}-Q_{1}}{V_{2}-V_{1}}$$

The IC curve analysis shows that the peak shift indicates the change in platform potential, the insertion and removal resistance of lithium ions increases, and the peak area represents the capacity charged or released during the phase transition process. The peak point represents the phase transition point of the material [35]. After differential processing of the battery data, it can be clearly seen that the curve peak value, peak area, and peak voltage change with the increase in the number of cycles. Therefore, Figure 3b reveals that the peak of the ICA curve, the peak area, and the voltage corresponding to the peak are often used as HI alone or in combination with different estimation methods to estimate the battery’s state of health. For example, in [36], by extracting HI from four different methods, the authors found that the peak, peak valley, and trough of the IC curve and their corresponding voltage were the best-estimated effects when the HI was used, and the RMSE was only 0.2%. In [37], the author extracted the peak value of the IC curve, the corresponding voltage, the energy, and the capacity of the constant current charging interval determined by the peak value of the IC curve as HI and established a multi-feature combination HI, which can better reflect the degree of battery degradation compared with a single HI. Ye et al. [38] performed a slight overcharge voltage cycle on the battery and used IC analysis to obtain the characteristic peak value, which was used as an HI to achieve an accurate estimate of SOH. In addition, Wang et al. [39] considered the IC curves of different charge and discharge rates. Finally, they used the 1/6C data for ICA to estimate the SOH of the batteries with varying levels of aging. Zhou et al. [40] also studied the SOH of fast-charging LIB through ICA and found the difference between the IC curve of the fast-charging battery and the battery at a lower charge rate. The peak with a noticeable change was extracted as the HI to estimate the SOH of fast-charging batteries, and the average error was reduced by more than 90%. Guo et al. [41] proposed a five-point moving window technique to obtain the IC peaks of multiple voltage intervals, and the robustness of the estimated results was achieved by using this characteristic quantity. She et al. [42] obtained the peak of the IC curve by filtering to evaluate the aging state of the battery based on a large number of real-time operating data, providing a new idea for the aging prediction of the actual use of the battery. In [43], Fan et al. treated noise interference based on Gaussian filters using four groups of HIs extracted from IC curves and confirmed that the selected HI was highly correlated with capacity degradation through Pearson correlation analysis. Huang et al. [44] used data reconstruction to process the data, reduce the influence of noise, and extract multiple view features such as the maximum value, the corresponding voltage, and other values around the maximum value from the obtained IC curve. This method improves the sensitivity of SOH estimation. Data from charge and discharge cycles at different magnifications can also be used for IC analysis, and the correlation between IC peak and SOH changes can also be expressed [45]. It can be seen that the HI extracted from the IC curve has a strong correlation with SOH, which performs well in estimating the SOH of LIB and also improves the robustness of the estimated results [46,47,48,49,50,51]. In addition, when the battery voltage reaches a constant value, dQ/dV reaches a local maximum near full charge, which can be used as a criterion indicating that the battery is full and not overcharged.

The expression of DVA is shown in Equation (2); similar to ICA, DVA also differentiates the capacity and voltage of the battery, but switches the numerator and denominator:

(2) $$\frac{dV}{dQ}=\frac{V_{2}-V_{1}}{Q_{2}-Q_{1}}$$

**Figure 4:**
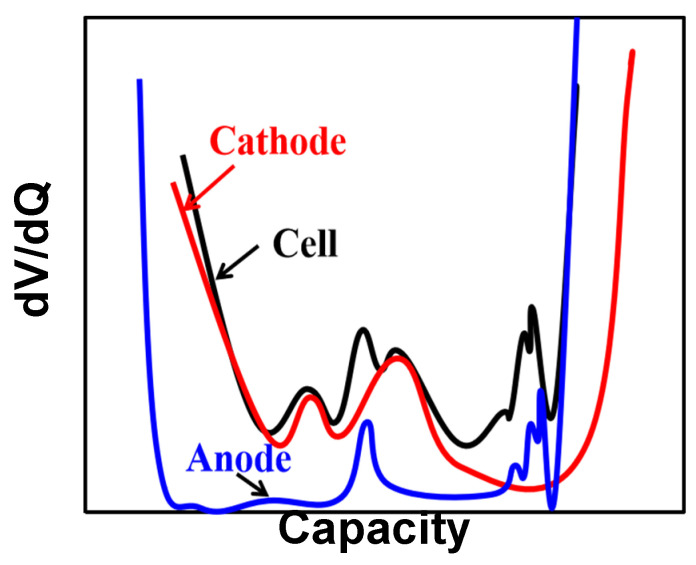
The mapping between full cell and half cell.

DVA analysis can explore the changes in the process of lithium insertion or dilithium of the active material inside the battery, which is often associated with the aging mechanism of batteries. In the DV curve, the contribution of the positive and negative poles can be distinguished by making a half cell. The voltage capacity data of the positive half cell and the negative half cell are obtained by small-current charging and discharging tests, respectively. On the basis of the volt–capacity curve, the corresponding relationship is obtained by coordinate conversion and displacement. After comparison, the DV processing of voltage and capacity is plotted to obtain the DV curve shown in Figure 4. It can be seen that the DV value of the battery is relatively large at the beginning and end, which is because the internal resistance of the battery is small in the early stage of charge and discharge, the current flows smoothly, and the reaction rate is fast, resulting in a higher differential voltage at the initial voltage and the final voltage. The cathode and anode each have peaks corresponding to the full battery, which is of great significance for understanding the aging mechanism of the battery. In [52], the author suggests that the presence of a peak in the DV curve indicates the formation of a new single phase in the graphite electrode, and the width of the peak corresponds to the maximum amount of lithium embedded, so the change in the peak width in the DV can be used to assess the loss of active materials in the negative electrode. In [53], the aging mechanism of the 18,650 battery was quantitatively studied using the DV analysis method. An essential assumption of the dV/dQ method is that the distance between the peaks is proportional to the capacity of the active material. Therefore, the distance between the peaks of the aged batteries shows that the losses of positive active material and lithium ions are the main factors leading to the battery’s aging.

In the same way, a sharp temperature rise can be observed shortly before the battery runs out and, thus, is an early indicator of an upcoming deep discharge and exhaust heat event at dV/dQ peaks [54]. Adam et al. [55] used differential charging voltage analysis to detect lithium deposition and found that the peak value at a high charging ratio represented the beginning of lithium deposition. Zhang et al. [56] combined the Coulomb counting method with DVA to estimate the SOH in the constant discharge phase. Wang et al. [57] used the interval between two inflection points in the DV curve to estimate SOH, established a linear regression between the DV curve transformation parameters and SOH, and achieved an estimate of the SOH of the battery pack within the error range of 2%. At the same time, the author also compares CV and DVA techniques, deduces the equivalent relation, and introduces the local data symmetry point method to calculate the DV curve, which is smoother than the DV curve obtained by numerical derivation [58]. Since the DV curve can monitor and quantify the aging mechanism of LIBs, Maitane et al. [59] estimated the SOH of LiFePO_4_ battery packs based on the characteristics of the DV curve. They discussed the application of this method in BMS systems. Zheng et al. [60] used natural interpolation to derive SOC-DV models from battery packs with different aging levels combined with the Kalman filter and particle filter. They estimated the SOC of the battery with a small error. Using the regional capacity in the DV curve as the HI, Zhou et al. [61] propose an online SOH estimation method based on this HI and inconsistent LiFePO_4_ battery packs, which reduces the computational burden and has an error of less than 2% in the estimation of eight cell modules. The algorithms or methods used in the mentioned literature and their advantages are briefly summarized in Table 2.

#### 2.2.2. Correlated with Resistance Increase

Lithium-ion batteries in the cycle process, in addition to capacity attenuation, also lead to an increase in battery resistance, so in the battery SOH estimate, it is an excellent choice to start with the rise of battery resistance. Electrochemical impedance spectroscopy (EIS) has proven to be an effective method to estimate the SOH of lithium-ion batteries. By establishing the relationship between changes in battery SOH and equivalent-circuit-model (ECM) parameters, the parameters are used to estimate SOH effectively. At the same time, the parameters can be linked with the aging mechanism of the battery to understand the aging process better. With the aging of the battery, the irreversible loss of lithium ions reduces the chemical reaction rate of lithium ions and the charge transfer process is more complex, so the charge transfer resistance is significantly increased; thus, it can be considered that the charge transfer resistance is related to the loss of lithium ions [62]. The growth, decomposition, and regeneration of a solid electrolyte interface film increases R_SEI_, which is also associated with the loss of lithium ions (LLI) [63]. Through extensive test analysis in [64], the author found that the charge transfer resistance underwent a significant aging change with the aging of the battery and showed an excellent ability to resist SOC drift and external resistance changes; thus, it could be used as an HI and reduced the SOH estimation error. In addition, the charge transfer resistance and solid-phase diffusion coefficient were extracted based on the electrochemical model as the internal HI to estimate SOH, which could achieve high-precision battery state estimation according to the internal reaction mechanism of the battery [65]. Bi et al. [66] developed a new second-order equivalent circuit model for battery packs. They applied the change in internal resistance identified by the model to the dynamic estimation of SOH when the accuracy of the equivalent circuit model of battery packs was not high.

While other external conditions remain the same during battery use, the internal resistance increases, and the battery capacity decreases. The variation in resistance means that the voltage response of the current pulse is not constant, and more specifically, the voltage responses of the different current pulses contain information about the SOH of the battery. After the hybrid pulse power characteristic (HPPC) test of the battery, it can be observed that its voltage and current change with the aging of the battery, and the specific resistance value is obtained through the calculation of DC internal resistance, which can be used as an effective HI. For example, in [67], the researchers extracted features from the voltage response of the charge/discharge current pulse as an HI, the inflection point in the voltage response curve, by building an SVR-based estimator; the results show that the estimation error of the proposed method was 0.0017. Similarly, sensitive points of voltage response extracted from short-term current pulse testing can also be used as HIs, and this is easier to realize in practical applications [68]. The models and methods used in HI literature related to resistance increases and their respective advantages are summarized in Table 3.

#### 2.2.3. Correlated with Temperature

The temperature change also has a particular impact on the battery’s aging. Therefore, by processing the temperature, the extracted signal can also be used to estimate the SOH of the battery. Differential thermal voltammetry (DTV) was shown to identify aging similarly to ICA. This technology can avoid errors caused by temperature changes. Only voltage and temperature data during constant current charging or discharging must be differentiated to analyze aging mechanisms such as LLI, LAM (loss of active material), and capacity loss [69]. DTV also shows the peak value by processing similarly to ICA. The peak value represents the area in the discharge curve that generates much heat, but the voltage change is not apparent. The transition between the peaks represents the phase transition process of a particular electrode. In [70], the author chooses the most significant positive peak value as HI because the most considerable positive peak value has a clear and consistent change in the aging process. After processing with the same method, the researchers extracted the peak value, peak valley, and peak position in the DTV curve as HIs, analyzed them using the Pearson correlation analysis method, identified four HIs strongly related to the capacity decline trend, established the battery degradation model, and estimated the SOH. The model based on GPR has the best accuracy and robustness, and the maximum relative error of the estimate is within 2% [71]. In addition, the index extracted from the curve of the temperature rate of change can also be used to estimate the SOH. In [72], Wu et al. obtained the distance between the cooling areas by measuring the battery surface temperature during the constant current charging stage, defined as tin. The HI and SOH were linearly correlated, and the correlation coefficient was 0.954. By analyzing the temperature variation within the voltage variation range, the temperature at which the voltage reached 3V was defined as T1. When the voltage reached 3.8V, it was T2, and the proposed characteristic TIEDTD was equal to T2–T1. The Pearson and Spearman correlation analysis found that TIEDTD was negatively correlated with the trend of capacity change [73]. Zhang et al. [74] converted the battery voltage and temperature data obtained from aging experiments into images by encoding, visually representing the subtle changes in the data. They combined the direct data, such as voltage and temperature, with the model, showing great potential for estimating SOH. Wu et al. [75] established a variety of batteries with different temperatures and aging degrees, used the function to calculate the probability of a single model, and fused it to obtain state estimation results; experimental verification proved that the method could accurately track SOC. In addition, in [76], Zhang et al. extracted five HIs based on three curves of surface temperature, ICA, and DVA, respectively, as input for the model and combined all features with a weighted average method to predict SOH, reducing the calculation burden and improving the accuracy of estimation. Tian et al. [77] established a relationship between the partial temperature difference curve and SOH over the voltage range, considering the temperature change, and found that the estimation of SOH based on temperature difference has less computational burden and more minor root mean square error compared with the ICA method. Mejdoubi et al. [78] discussed the effectiveness of estimating SOH and SOC at different operating temperatures and discharge currents based on changes in the battery surface temperature. Li et al. [79] proposed that the enhanced coulomb count is based on surface temperature characterization, which improves the accuracy of SOC estimation. Similarly, Huang et al. [80] also took into account the rapidly changing temperature to accurately estimate the SOC of the battery based on the model. To avoid nonlinear degradation of the battery capacity, Jia et al. [81] processed the temperature characteristic data in the frequency domain. They predicted the SOH by considering the variation in battery discharge temperature. In Table 4, the models and methods of HI research related to temperature are summarized.

**Table 3:** Based on the resistance.

Reference	HI	Algorithm/Method	Advantage
[64]	Charge transfer resistance	ECM	The HI has a good anti-SOC drift ability
[65]	Charge transfer resistance and solid phase diffusion coefficient	RF ANN	It has high estimation accuracy in different scenarios
[66]	Change in internal resistance	GPF Second-order equivalent circuit model	Demonstrated reliability on the actual electric vehicle data
[67]	The inflection point of the voltage response curve in the current pulse	SVR	The estimated error is only 0.0017
[68]	Sensitive point of voltage response in short-term current pulse test	SVR Genetic algorithm	The data acquisition process is easy and efficient

**Table 4:** Based on the temperature.

Reference	HI	Algorithm/Method	Advantage
[70]	Peak value of DTV curve	/	Less sensitive to temperature introduction error
[71]	Peak value, peak valley, peak position	GPR	It has high estimation accuracy and robust performance
[72]	The battery indicates the temperature of the cooling zone distance	/	The strong linear relationship between HI and SOH provides reliable support for online SOH estimation
[73]	Temperature change within the range of voltage change	MKSVM	Has higher SOH prediction accuracy for LIB
[74]	Subtle changes in voltage and temperature	Long Short-Term Memory Networks	Demonstrated that direct data such as voltage and temperature have great potential in predicting the SOH of batteries
[75]	Decoupling temperature	IMM	Accurate SOC tracking and real-time capacity estimation are realized
[76]	Surface temperature ICA and DVA	WOA-Elman	The calculation burden is reduced, and the estimation accuracy is improved
[77]	Partial temperature difference curve in the voltage range	SVR	The accuracy of estimation is improved, and the computational burden is negligible
[78]	Battery surface temperature changes	EKF	It shows the effectiveness of SOC and SOH estimation under different temperature and discharge current conditions
[79]	Surface temperature characterization	DTW	Effectively improves SOC estimation accuracy under various temperature conditions
[80]	Rapid changes in temperature	EKF	Describes the battery pack’s dynamic behavior well, improving the estimation accuracy
[81]	Temperature change during battery discharge	Wavelet neural network Ensemble learning	Overcomes nonlinear fluctuations in battery capacity degradation

#### 2.2.4. Other HIs

**Figure 5:**
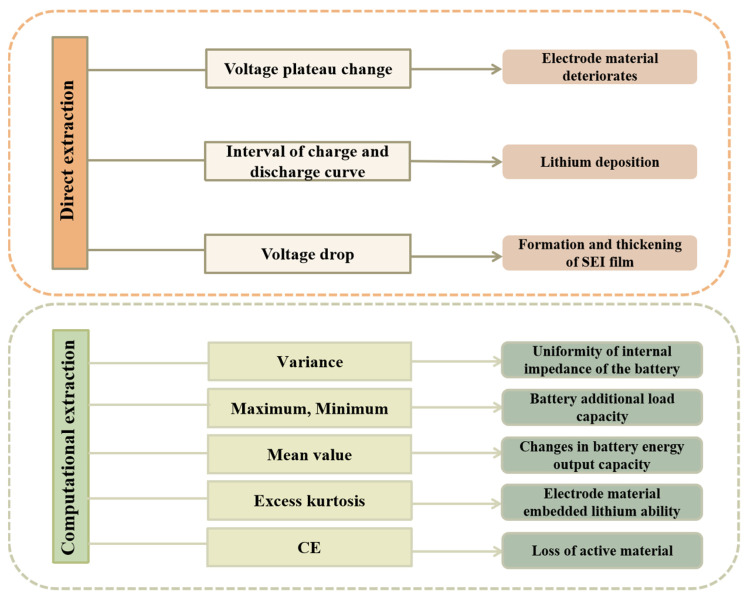
Other HI correspondences with aging mechanism.

In the examination of HIs utilized for estimating the SOH of LIBs, it is important to note that, in addition to the three previously mentioned categories of HIs, there is a diverse array of additional indicators that significantly enhance SOH estimation. These indicators can be classified into two main categories, as illustrated in Figure 5: HIs that are directly extracted during the charging and discharging processes and HIs that are derived by calculating characteristic quantities throughout these operations. We will discuss each of these categories in detail.

In practical applications, the charging and discharging processes of batteries are often random and influenced by a variety of external factors, including the driver’s habits, ambient temperature, and road conditions. Consequently, numerous studies have focused on investigating the decay mechanisms of batteries by analyzing partial charge and discharge curves. The goal of these studies is to extract HIs that exhibit a strong correlation with the SOH of the battery, thereby enabling more accurate SOH estimates.

As a battery ages, observable changes in the voltage profile during the charging and discharging processes can indicate degradation of the electrode material. Additionally, an increasing gap between the charging and discharging curves may suggest that the lithium ion embedding and de-embedding processes within the electrode are becoming impeded, potentially signaling issues such as lithium deposition. Furthermore, the formation and thickening of the SEI (solid electrolyte interface) film can lead to increased internal resistance, resulting in a greater voltage drop. Therefore, by meticulously analyzing the charge and discharge curves, we can extract valuable insights into the aging mechanisms of lithium-ion batteries, facilitating effective estimates of their SOH. Table 5 shows a large number of other HI research models and methods.

Li et al. [82] extracted HIs from the partial charge curve and found that the accuracy of the estimation was higher for the partial charge curves of lower starting voltage, larger charges, and large sliding windows. Wang et al. [83] used the current at the CV charging stages as an external feature to estimate SOH because the maximum curvature of the current curve decreased as the battery aged. In the random charging scenario, Shen et al. [84] extracted the SOH-related HI by analyzing the distribution of charging voltage. They adopted the extreme learning machine algorithm to achieve a reliable estimate of the SOH in the short-term random charging scenario. Meng et al. [85] divided the constant current charging stage into several short segments and extracted HI from each short segment according to the charging capacity and actual charging voltage; the estimation of the short segment also has good robustness. In addition, in [86,87,88,89,90,91,92], the researchers used the characteristic quantity extracted from the partial charge/discharge data as the HI. They achieved good performance in estimating the SOH of the LIB. The concepts of regional frequency and regional voltage were also applied to the estimation of the SOH of the LIB, and Huang et al. [93] found that SOH is a simple linear function of regional frequency, with R^2^ fitting to the appropriate regional voltage and reaching more than 0.99. Song et al. [94] extracted HIs based on the charge and discharge rate, discharge of depth, and temperature in historical data, and estimated the SOH of LIBs using a big data platform, with a maximum relative error of 4.5%. Based on the data collected during the charging and driving phases, including variables such as speed, temperature, and charging power as HIs, Mawonou et al. [95] introduced the random forest algorithm to predict battery aging, generating an estimated error of only 1.27%. Due to the diversity and complexity of practical scenarios, extracting HIs from electromechanical characteristics to estimate SOH is also a good choice. Gong et al. [96] extracted HIs directly from the stress curve and used correlation analysis to select the most appropriate HI for an accurate estimation. Moreover, energy could also be selected as an HI. Gong et al. [97] extracted energy at the constant current charging stage, constant voltage charging stage, and equal discharging voltage interval as HI and evaluated the applicability and prediction accuracy of the SOH estimation model based on the above energy characteristics for different lithium-ion batteries in different charging and discharging scenarios.

Another approach involves calculating characteristic quantities during the charging and discharging processes and using summary statistics to analyze changes in the shape and position of the battery’s charge and discharge curves. For instance, the voltage curve can be transformed into various mathematical statistical measures, such as variance, maximum value, minimum value, and average value. The calculation formulas are summarized in Table S1 of the Supplementary Materials. By analyzing these HIs, we can achieve a more accurate estimate of the SOH of the battery. These features also contain significant information regarding the aging mechanisms of lithium-ion batteries. Among these statistics, variance reflects the stability of current or voltage fluctuations; a higher variance indicates that the internal impedance of the battery is uneven. This non-uniformity is often associated with uneven chemical reactions within the battery and the degradation of electrode materials. Meanwhile, the maximum and minimum values can reveal reductions in the battery’s additional load capacity and highlight potential safety hazards, such as overcharging or overheating. Changes in the average value indicate alterations in the battery’s energy output capacity, which may be linked to electrolyte decomposition. Additionally, an increase in excess kurtosis typically signifies a rise in polarization phenomena, reflecting a decline in the lithium insertion capacity of the electrode material.

In [98], the author used a data-driven method to extract HIs based on the relaxation voltage curve and converted the relaxation voltage curve into six statistical features: variance, skewness, maxima, minima, mean, and excess kurtosis. Among them, the RMSE of the optimal base model is found to be 1.1% by analyzing the selection of Var, Ske, and Max as inputs to predict the capacity in the next cycle. Similarly, in [99], the author calculated the summary statistics of the curve for each cycle, such as the minima, mean, and variance, to capture the change in the voltage curve between cycles and conclude that the loss of anode active material causes the change in discharge voltage and causes the anode capacity to eventually fall below the remaining lithium ion inventory. Dai et al. [100] extracted a series of data from the battery test curve as the initial HI, calculated the six statistical features of each initial HI (i.e., mean, median, lower quartile, range, upper quartile, and standard deviation), and analyzed the optimal HI set by different combinations of these statistical features, which showed good SOH estimation performance on different LIBs. Liu et al. [101] found that the constant current charge curve changed slightly as the cycle progressed, and the coefficient set of the analytic expression of the curve fitted with a polynomial of the same order could be used as an HI. After considering the influence of the charge rate, the logarithmic charge curve was obtained using the logarithmic processing method. With the analytic coefficient set fitted by a logarithmic charge curve as input, the SOH of the battery was estimated by using a linear regression algorithm. The effectiveness of the SOH is often estimated to depend on the correlation between the selected features and the SOH. Therefore, Sui et al. [102] extracted the fuzzy entropy of battery voltage as a new kind of HI and verified the effectiveness of the HI through experimental results. Goh et al. [103] proposed a new method of HI extraction based on the U-string curvature model, segmented the discharge stage according to the curvature of the discharge curve, selected the HI highly correlated with the SOH at the discharge platform stage, used different machine learning algorithms to estimate the SOH, and verified the availability of the HI. However, due to the computational burden and other problems with extracting the HI from the charge and discharge curves, Mao et al. [104] proposed a new HI called equal voltage range sampling count number to estimate the SOH, which reduced the calculation amount of BMS and ensured accuracy in the estimation. Liu et al. [105] use the voltage variance over equal time intervals as a new HI extracted from various voltage indicators, and this was flexible for online estimation. LIBs are faced with many uncertainties if they want to obtain efficient eigenvalues in actual use. Therefore, Huang et al. [106] proposed a SOH estimation method based on the local coulomb count curve by calculating the coulomb quantity of the local voltage segment, verifying eight batteries under different current conditions, and proving the estimation accuracy of the method. The continuous development of data extraction and analysis technology has also revealed a new idea of using big data to estimate SOH. Usually, in the battery cycle, high coulomb efficiency (CE) means that the battery cycle life is long, so the study proved that CE can also be used to predict the aging of LIBs. Yang et al. [107] found through research that a sharp decline in the CE curve can be associated with an inflection point warning of accelerated aging in batteries, and that the decline in the CE value is caused by the LAM. The semi-empirical model derived by Yang et al. [108] based on coulomb efficiency can effectively capture the degradation trend of LiFePO_4_ batteries, simulate the use of batteries in real life, and carry out an online assessment of SOH.

**Table 5:** Other HIs.

Reference	HIs	Algorithm/Model	Advantage
[82]	Partial charging curve	MLR, GPR, SVR	Accuracy is higher
[83]	Current at CV charge stage	First-order ECM	There is a robust linear relationship between HI and battery capacity
[85]	Charging capacity and charging voltage	Kernel ridge regression	The short segment has good robustness
[93]	Regional frequency and regional voltage	PDF	To overcome the poor SOH estimation results caused by inconsistencies in modules
[94]	Charge and discharge rate, DOD, temperature	Big data platform	Big data makes it easier to realize the intelligence of SOH estimation
[95]	Speed, temperature, charging power	Random forest	It provides a reference for LIB rapid aging diagnosis in electric vehicle applications
[97]	The energy at CC, CV charging stage	GPR	It has high estimation accuracy and generalization ability
[98,99,100]	Convert voltage curves to statistics: variance, skewness, maximum, minimum, mean, and excess kurtosis	ZSL	The results show that the relaxation voltage estimation capacity is successful
[101]	The analytical coefficient set is fitted to the constant current charge curve	Linear regression	Small computation, suitable for online estimation
[102]	Fuzzy entropy of battery voltage	SVR	The accuracy of SOH estimation based on temperature change is improved
[103]	Electromechanical characteristics	LSTM, ANN	This HI has good accuracy, reliability, and robustness
[104]	Equal voltage range sampling count number	GPR	Reduced the calculation amount of BMS
[105]	Voltage variance over equal time intervals	ELM	Achieved flexibility for online estimation
[106,107,108]	CE	Particle filtration	It helps develop diagnostic models for battery aging, showing higher predictive accuracy.

### 2.3. Summary

To sum up, there are numerous HIs to choose from, and below, we provide a concise summary of various HI scenarios. First, HIs associated with capacity decay are suitable for assessing the overall health of the battery after long-term use. These indicators require regular charge–discharge cycle testing to obtain accurate capacity data, making them ideal for evaluating the battery’s condition over extended periods. By using voltage and current sensors, the voltage of the battery is measured, the current is monitored, and the charging and discharging state of the battery is reflected in addition to the power output and input. HIs related to the increase in internal resistance are applicable to scenarios involving frequent charge and discharge cycles. Therefore, they are highly beneficial for real-time monitoring of battery SOH in vehicles and energy storage systems. On-board sensors and real-time data acquisition systems can perform online detection to obtain the relevant HIs. Using the impedance measured by EIS as an HI allows for detailed analysis of the battery’s internal state, particularly the characteristics of the electrolyte and electrode interface. This is suitable for fault diagnosis and early warning of battery issues. If the effects of electrolyte decomposition and loss of active materials need to be assessed, open-circuit voltage (OCV) can be used as an HI to evaluate the aging state of the battery. This is particularly suitable for SOH estimation when the battery is at rest. For HIs related to temperature changes, they are suitable for monitoring the thermal management performance of the battery in high-power applications. With the use of temperature sensors, we expect to obtain more accurate data. This helps to evaluate the battery’s performance and safety at different ambient temperatures.

When statistical characteristics are used as HIs, they correspond to a variety of application scenarios. For example, the average value of the calculated voltage is suitable for monitoring the SOH of the battery in a stable working state, especially under constant load conditions. Variance, which may indicate inconsistencies or faults within the battery, such as uneven distribution of electrode materials or electrolyte decomposition, is suitable for estimating the stability of the battery in complex environments, such as temperature changes or load fluctuations. Abnormal changes in the maximum and minimum values can indicate the risk of thermal runaway, allowing for the detection of overcharge and overdischarge under extreme conditions. The kurtosis of the voltage distribution can indicate a transient fault within the battery, such as a local short circuit or poor contact.

Different health factors are suitable for different use conditions and application scenarios. Selecting appropriate health factors and conducting a comprehensive evaluation of multiple health factors can provide a more thorough and accurate assessment of battery health. LIBs, as a mainstream battery type, play a vital role in electric vehicles and other fields. Due to the complexity of accurately estimating their SOH, this task is also urgent. From the current research on extracting HIs to estimate the SOH of LIBs, it is evident that many HIs can be extracted during battery operation. Through the processing of primary data, the extracted HIs are more closely related to the SOH of the battery, leading to more accurate SOH estimates. These HIs extracted from primary operating data are more sensitive to battery aging. However, in the pursuit of a more accurate SOH estimate, single HIs cannot fully describe battery aging, and the feasibility of various HIs in other types of batteries needs further investigation. Therefore, the importance and urgency of developing comprehensive SOH estimation methods that fuse multiple HIs are evident. Future studies should further explore the fusion of multi-modal health factors and multi-parameter comprehensive evaluation methods to enhance the reliability and accuracy of battery health status assessment.

## 3. SOH Estimation Model

### 3.1. Model-Based Method

#### 3.1.1. ECM-Based Method

**Figure 6:**
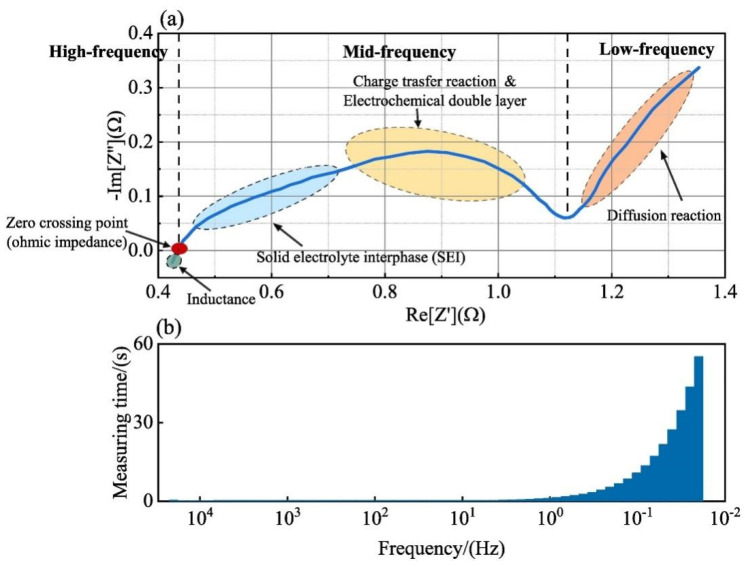
(**a**) Schematic of electrochemical impedance spectrum. (**b**) Measurement time of impedance at different frequencies [126].

The ECM model simulates the battery’s performance by constructing a circuit with electrical components such as resistance, capacitor, voltage source, and current source in the circuit. Since the ECM model is an experience-based approach, relevant experimental data are needed for parameter fitting. Moreover, the ECM models have the advantages of small computation and high solving efficiency, and the RC parallel structure exists in the equivalent circuit, which can follow the condition of drastic load change well. The research on lithium-ion battery equivalent circuit models is constantly developing, mainly the Rint, Thevenin, second-order RC, PNVG, and GNL models [109]. The specific representations and disadvantages of each model are shown in Table 6. The HI of the SOH estimated by the ECM model is usually selected in Section 2.2.2, related to the increase in resistance. For example, the ECM model can construct the relationship between lithium-ion battery resistance and SOH under different temperatures and SOC [110,111]. EIS data are obtained by ECM, and EIS is used to analyze the charge transfer in the electrochemical process by measuring the excitation and response of the AC electrical signal, in which the analysis of the spectrum can reveal the dynamics and electrode interface structure information of the electrochemical process. As shown in Figure 6, the Nyquist diagram of the battery can be divided into three parts: high-frequency, medium-frequency, and low-frequency, which are mainly composed of two semi-circles and a straight line. The high-frequency region is the kinetic control of the electrode reaction. The first semi-circle in the medium-frequency region is dominated by the impedance of the SEI film, and the second semi-circle is the impedance caused by charge transfer and charge accumulation in the double electric layer. The low-frequency region involves the electrode reaction of the reactant or product diffusion process [112,113]. By fitting the data of EIS, the specific value of resistance can be obtained, which can be used as an alternative to HIs. However, due to the various internal reaction mechanisms of other lithium-ion batteries, the applicability of the ECM method is limited. To solve this problem, Li et al. [114] improved the ECM to improve the fitting performance of EIS data and better reflect the internal reaction process of batteries. A new method was proposed to estimate the SOH of batteries by combining SEI film resistance and charge transfer resistance. Compared with traditional ECM methods, MLECM has higher estimation accuracy and involves less computation [115].

In the research of SOH estimation methods based on the ECM model, it has been proven that this technology has good engineering application value [116]. Xu et al. [117] improved the Thevenin ECM according to the characteristics of LiFePO_4_ batteries and proposed a new online SOC estimation method. The improved model can accurately estimate the SOC even when there are errors in the current measurement. The auto-regressive ECM proposed by Liu et al. [118] showed high estimation accuracy and robustness for joint estimation of SOH and SOC. Liu et al. [119] used a fractional-order-based ECM to estimate the SOP of LIB. Since the estimation of the SOP generally assumes that the battery operates under extreme operating conditions, the fractional-order-based model can improve the accuracy of the estimation and reduce the influence of the operating conditions. Based on the Rint model, Wu et al. [120] proposed an estimation method of SOP based on two assumptions, which have small MAEs under different working conditions and temperatures. Amir et al. [121] used a 2-RC model to capture the degradation of LIB, which is time-dependent and can also track the effect of temperature on battery aging, resulting in a more accurate estimate of SOH. Naseri et al. [122], based on wiener structure, enhanced ECM to estimate the nonlinear performance of LIB. Compared with 2-RC ECM, this method improved the estimation accuracy of SOC by 1.5% and had higher computational efficiency. Chen et al. [123] proposed an integrated ECM and data-driven approach for SOH estimation, which was validated on different battery datasets and showed better convergence and generalization. Xu et al. [124] modified the ECM model and then combined it with electrochemical mechanisms and diffusion processes to jointly estimate the state of LIB, showing better accuracy and stronger robustness in predicting future states. Huo et al. [125] considered the effect of temperature on battery performance and proposed an improved ECM based on a 2-RC model that can study the effects of ambient temperature and battery surface temperature. Compared with traditional ECM, this model improves the estimation accuracy of the SOC under different operating conditions.

#### 3.1.2. Electrochemical Model

**Figure 7:**
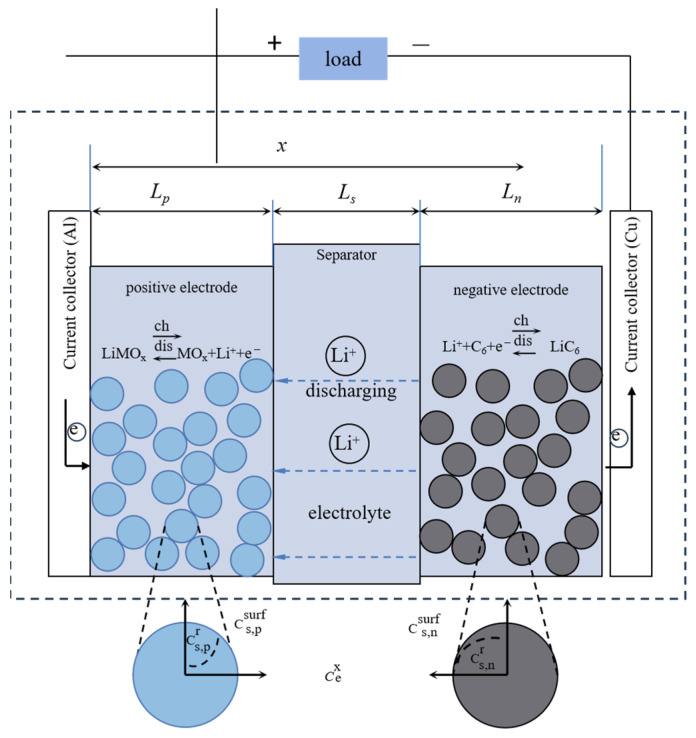
The lithium-ion battery P2D model.

**Figure 8:**
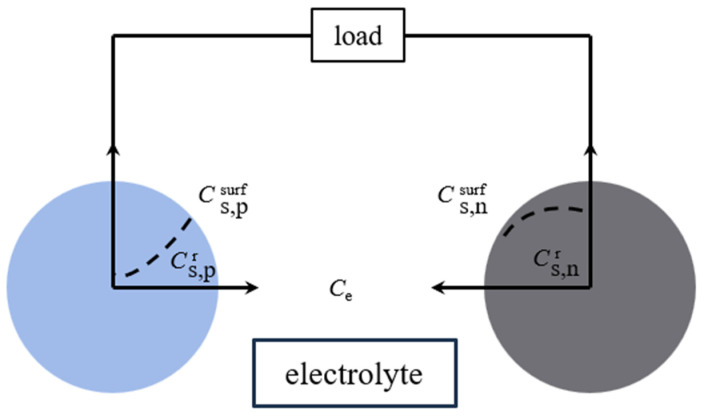
Schematic of single-particle model.

Based on the porous theory and the concentrated solution theory, the electrochemical model describes the charge and discharge behavior of lithium-ion batteries from the perspective of the electrochemical mechanism by numerically simulating the internal electrochemical reaction kinetics, mass transfer, heat transfer, and other micro-reaction processes. The electrochemical models of lithium-ion batteries mainly include a single-particle model, a pseudo-two-dimensional (P2D) model, and a simplified pseudo-two-dimensional model. The most classical electrochemical model is the P2D electrochemical model proposed by Doyle [127], as shown in Figure 7, which has two dimensions, one thickness, and one radial dimension. The model equates the lithium-ion battery to an electrode composed of numerous spherical solid particles (positive and negative) and a sandwich structure consisting of a diaphragm and electrolyte. Fick’s diffusion law was used to describe the concentration distribution of lithium ions in the solid-phase particles of the electrode. The lithium-ion concentration in the electrolyte and the diaphragm was calculated based on the law of conservation of charge and conservation of matter, the electric potential in the solid-phase electrode was calculated based on Ohm’s law, and the liquid phase electric potential in the electrolyte and the diaphragm was calculated based on Kirchhoff’s law and Ohm’s law. The Butler–Volmer formula calculated the electrode reaction kinetics [128,129,130]. The single-particle model in Figure 8 was obtained by simplifying the P2D model, the simplest electrochemical model for lithium-ion batteries. In the single-particle model, two spherical particles represent lithium-ion batteries’ positive and negative electrodes. Lithium ions’ embedding and removal processes are assumed to occur on the spherical particles, and the electrolyte concentration and its internal potential are considered constant [131].

Due to the superiority of EM in inherent physical representation, it is often used by researchers to estimate the SOH of LIB [132]. Other methods, such as order reduction, are used to improve EM to improve the accuracy of estimation. For example, Hosseininasab et al. [133] used a reduced-order electrochemical model to assess the SOH of LIB, which was not very computationally costly due to the small number of calibration parameters, and the authors validated the effectiveness of the method at different aging levels. Li et al. [134] combined a reduced-order electrochemical model with a double nonlinear filter to achieve high-fidelity estimates of SOC and SOH over the entire life of a battery. Wang et al. [135] used the pseudo-spectral method to solve the solid-phase diffusion equation and the Galerkin method to simplify the liquid-phase concentration equation, which improved the shortcomings of the existing order reduction method, such as the large calculation amount and small application range, and showed good performance in SOC estimation. Fan et al. [136] also used the Galerkin method to reduce the order of the model, using the reduced order diffusion model in liquid and solid phases to develop an extended single-particle model including electrolyte dynamics, which has high simulation speed and good performance in long-life simulation, control, and estimation applications. Xiong et al. [137] identified the aging state of LIB based on EM instead of traditional ECM and black box models, established mathematical equations to describe the physical and chemical behavior of the battery based on electrochemical theory, and estimated the error range of SOH to be limited to 3%. Xu et al. [138] described the distribution of lithium content inside a battery using a minimalist EM that related the SOH to the attenuation of capacity due to the irreversible loss of lithium ions. Lim et al. [139] introduced a simple reduced-order EM to accurately predict SOH under various calendar aging conditions. Li et al. [140] verified the estimation accuracy of an extended single-particle model-based LIB state observer with high-fidelity EM, demonstrating that all estimated states presented high schedules and robustness for real-time applications. In addition, Tang et al. [141] developed a thermoelectric model for LIB, comprising a coupled system of partial and ordinary differential equations that can be used to estimate SOC and track average temperature while improving estimation accuracy. Verma et al. [142] presented an airborne reduced-order electrochemical thermal model of a composite cathode battery pack with good accuracy for SOC and voltage prediction under both static and dynamic loads.

It can be seen that this model-based approach is widely used in estimating various states of LIB. Through these models, we can better understand the physical and chemical processes inside the battery and, thus, monitor the performance and condition of the battery. On the basis of deeply understanding the internal working principle and performance characteristics of the battery, it is essential to improve the safety of the battery with higher precision and stronger generalization of the estimation results, which is crucial to promote the development of electric vehicles, energy storage, and other fields, but also to provide more possibilities and opportunities for the progress of battery technology and application development.

### 3.2. Machine Learning (ML)-Based Method

**Figure 9:**
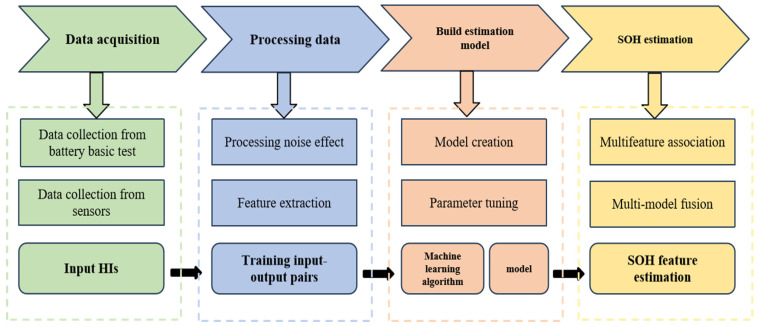
Workflow of machine algorithms.

ML is one of the most used methods in the process of using HI to estimate the SOH of the battery. Its workflow for battery aging modeling is shown in Figure 9. Various battery data are collected as input for model training. HIs are extracted, some ML is loaded, SOH is output, and the relationship between battery aging and SOH and HI is described. Finally, errors are calculated to compare the accuracy of various machine learning methods. ML methods can help design diagnostic models for battery aging [143], and in the estimation of SOH, a large number of studies have discussed linear regression, support vector regression (SVR), random forest, artificial neural networks (ANNs), Gaussian process regression (GPR), Bayesian estimation, and other methods [144,145,146]. This section focuses on an overview of the following four types of ML.

The GPR method is relatively simple and can be used in practical applications. Because the aging curves of lithium-ion batteries are non-linear and random, GPR is very suitable for simulating the data of lithium-ion batteries [147]. The GPR model provides a probability distribution of predicted values and can quantify uncertainty, which is important for the assessment of battery health. The model does not need to determine the specific form of the model in advance, and it can adapt to the complex data distribution. The predictions generated by GPR are generally smooth and suitable for working with continuous data. However, due to the high computational complexity of GPR, especially when the amount of data is large, the computation time and memory requirements will increase significantly. When using this model, it is necessary to select the appropriate kernel function and hyperparameter, which usually requires a significant amount of testing and verification, so most of the current research focuses on improving the GPR model to avoid these shortcomings. For example, Feng et al. [148] improved the variance function of GPR. They established SOH and RUL prediction methods for lithium-ion batteries based on GPR and polynomial regression, which have higher accuracy and robustness. Wang et al. [149] proposed a new estimated complex kernel function and selected different mean function and kernel function pairs to construct a GPR model. The MAE and RMSE estimated by this method are only 1.7% and 2.41%, respectively. The research also improves prediction accuracy by integrating multiple GPR models and developing various new strategies based on integrating GPR and other methods [150]. Combining DTV [151], IC [152], geometric impedance spectrum characteristics [153], charging curve [154], energy characteristics [155], thermal characteristics [156], and other different HI, a more reliable and more applicable estimation model can be built.

SVR builds regression models by finding support vectors in the training data and has certain advantages in SOH estimation because it can handle high-dimensional data [157]. However, many parameters need to be adjusted in SVR, which is sensitive to data. Therefore, these limitations should be overcome through appropriate parameter adjustment and data processing to improve the performance and generalization ability of the model [158]. Wu et al. [159] proposed a joint algorithm using Bat Algorithm and SVR to predict the SOH of lithium-ion batteries, overcoming the difficulty of SVR hyperparameter determination. Ben et al. [160] used a quantum-behaved particle swarm optimization (QPSO) algorithm to optimize ISVR kernel function parameters. Compared with classical SVR, the prediction results of this method are more reliable, providing early warning for SOH, and it is convenient for evaluating battery aging. Similarly, SVR can also be combined with the aforementioned HI extraction method to integrate the performance of multiple HIs, and after correlation analysis between extracted HI and battery capacity, SVR is used to build an estimation model. Through migration and fine-tuning, it can be applied to different cycle conditions and aging states, and it also has practical applications in real scenarios [91,161,162,163,164].

The neural network has good nonlinear mapping and self-learning abilities, which can contribute to the estimation of SOH [165]. The model can deal with complex nonlinear relationships and is suitable for highly nonlinear systems such as battery health state. The network structure can be adjusted to adapt to different data sets and tasks. However, the ANN model also has some shortcomings. For example, its internal mechanism is relatively complex, which makes it difficult to explain the decision-making process of the model. Without proper regularization and data enhancement techniques, ANN is prone to overfitting training data. Artificial neural networks (ANN) are widely used in battery management systems to monitor voltage, current, temperature, and other parameters; learn historical data; and identify battery capacity attenuation. It can improve the performance and reliability of battery systems [166,167]. Driscoll et al. [168] used the ANN model to estimate the SOH of the battery under different discharge conditions based on the HIs extracted from the voltage, current, and temperature curves. This model achieved efficient and accurate prediction and used less input data than other models. Using the HI mentioned in this paper as input, we could also obtain good SOH estimation results using the ANN model [169,170].

Random forest is an integrated learning algorithm that makes predictions by constructing multiple decision trees and synthesizing their results. It has the advantage of processing many features and data and is not easy to overfit when estimating the SOH of lithium-ion batteries [171]. Features based on random forests that can handle many features are often combined with other models. Randon forests can be used to select the best HI and then input these HIs into different models to make predictions, which can improve the accuracy and confidence of predictions. Yang et al. [172] used a convolutional neural network and random forest to estimate the SOH of lithium-ion batteries. They proved through experiments that the method can better use partial discharge data. Wang et al. [173] combined empirical mode decomposition, random forest, and a gated cycle unit, using the time intervals of constant rises and falls in voltage as His; applied deep learning and machine learning to battery health management; and provided a reference for battery health management and intelligent operation.

**Figure 10:**
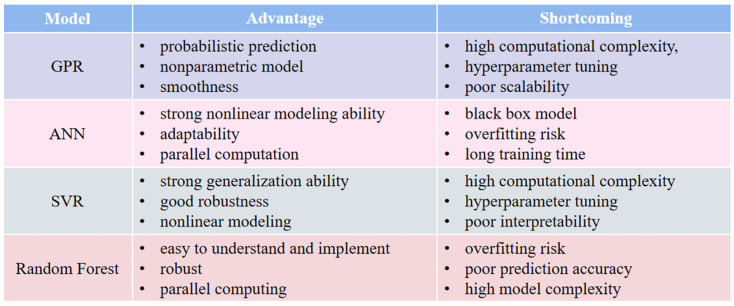
The advantages and disadvantages of different models.

In summary, machine algorithms are widely used in the estimation of SOH. Figure 10 summarizes the strengths and limitations of each of the four models. For probabilistic predictions and the quantification of uncertainty, GPR is a suitable choice. When dealing with intricate nonlinear relationships, ANNs are recommended. For robustness and strong generalization capabilities, SVR is preferred. If a straightforward and interpretable model is required, along with the ability to assess feature importance, random forests are the ideal option. They will continue to shine in future battery management systems due to their excellent performance in terms of computational effort, applicability, and accuracy of the results [174]. However, ML also faces some challenges in the estimation; the overall performance of the ML algorithm is affected by the accuracy of the data, and the aging process of lithium-ion batteries is complex, the determination of parameters is affected by various conditions, and the selection of hyperparameters should be performed carefully. Otherwise, it will increase the complexity of the work [175]. Therefore, based on using a single algorithm, multiple algorithms can be mixed to enhance the efficiency and robustness of estimation, and ensemble learning methods can be used to improve the training accuracy of the model [176,177]. In future studies, the performance of ML can be enhanced from multiple perspectives to strengthen its position in practical applications.

## 4. The Application Trend of Using HIs to Estimate SOH

### 4.1. Application in BMS

The future development trend of using HIs to estimate SOH primarily involves integrating multiple HIs into BMS. This approach enriches the data available to the BMS, thereby enhancing the system’s ability to accurately monitor and control battery performance. BMS is a critical component in electric vehicles, aircraft, and energy storage systems, tasked with monitoring, analyzing, and managing the health of lithium-ion batteries [178,179]. The system can monitor battery parameters such as voltage, temperature, and current in real time and predict battery life and performance degradation through advanced data analysis and algorithms. By leveraging these capabilities, users can promptly identify abnormal battery conditions, take timely maintenance actions, and extend the battery’s service life. Additionally, the BMS provides detailed health status reports and maintenance recommendations, helping users to better manage and maintain their batteries.

However, implementing the machine learning (ML) models described above in real-world battery management systems presents several challenges, both technical and operational. Below, we discuss some of the main challenges and their potential solutions.

#### 4.1.1. Data Quality and Quantity

High-quality data are essential for effective model training. However, in practical applications, data acquisition can be influenced by factors such as sensor accuracy, sampling frequency, and data integrity [180]. The annotation of battery health status requires specialized expertise, and the labeling process is often time-consuming and resource-intensive. Moreover, the diverse types of batteries and varying usage environments introduce significant complexity and variability into the data [181]. To address these challenges, we can enhance data integrity and accuracy by integrating data from multiple sensors; improve data quality using techniques such as filtering, denoising, and normalization; and augment data diversity and volume through methods like data synthesis and transfer learning.

#### 4.1.2. Real-Time and Computing Resources

BMS require real-time monitoring and prediction of battery status, which imposes stringent demands on the inference speed of the models. Embedded systems often have limited computational resources, making it challenging to run complex models efficiently [182]. To overcome these issues, we can reduce model complexity by employing lightweight architectures, utilize dedicated hardware to accelerate the model’s inference process, and offload a portion of the computational tasks to edge devices or cloud platforms to alleviate the burden on embedded systems.

#### 4.1.3. Interpretability and Transparency of the Model

Machine learning models are often characterized as black-box models, making it difficult to elucidate their internal mechanisms and decision-making processes [183]. This lack of transparency can erode users’ and engineers’ confidence in the predictions, especially in safety-critical applications. To mitigate this issue, we can employ interpretability techniques to provide insights into the model’s predictions. Additionally, integrating physical models with data-driven models can enhance the interpretability and transparency of the overall system, thereby fostering greater trust and reliability in the predictions.

#### 4.1.4. Generalization Ability and Robustness of the Model

To ensure that the model maintains robust performance across various types of buildings and usage environments, as well as handles outliers, noise, and missing data effectively, it is essential to enhance its generalization ability through multi-task learning [184]. Outliers and noise can be managed using data-cleaning and anomaly detection techniques, while the robustness of the model can be improved by employing ensemble learning methods [185]. These strategies collectively ensure that the model remains reliable and accurate under diverse and challenging conditions.

In summary, given the increasingly complex environments in which batteries are used, the lithium-ion battery health management system plays a pivotal role in enhancing users’ understanding of battery status, improving battery efficiency and safety, extending battery life, reducing maintenance costs, and bolstering system reliability and stability. As artificial intelligence continues to advance, the trend towards intelligent battery management systems is becoming increasingly evident. A key advantage of these systems is their capacity to learn from current data and adapt over time, thereby delivering accurate results even in complex scenarios [186]. Looking ahead, the integration of smart sensors into batteries will enable the detection of various internal signals that characterize health status, thereby enriching the input data for the battery management system and facilitating the broader application of machine learning models. This evolution will drive the progress and development of intelligent battery management systems, ultimately leading to more sophisticated and efficient battery management solutions [187].

### 4.2. Integration with Big Data

The integration of battery health estimation and big data platforms is increasingly exerting a significant influence on battery management systems (BMS). By leveraging big data technology, the assessment of battery health status becomes more precise and occurs in real time [188]. Big data platforms are capable of processing vast amounts of data from multiple sensors and devices, enabling the analysis of key parameters such as battery operational performance, charge and discharge cycles, and temperature variations for a more comprehensive health-monitoring framework. This convergence offers several notable advantages. Firstly, big data analytics can identify potential anomalous patterns in battery usage and predict impending failures in advance [189]. This early warning capability not only enhances battery safety but also optimizes operational and maintenance management, thereby reducing maintenance costs. Secondly, machine learning models grounded in big data can continuously learn and adapt from historical data, thereby improving the accuracy of battery health state estimations [190]. Lastly, the synergy between cloud and edge computing enables the BMS to achieve more efficient data processing and real-time monitoring, ensuring reliability even in complex and dynamic environments [191]. Looking forward, it is anticipated that the deep integration of battery health estimation and big data platforms will drive the intelligent and automated evolution of BMS. This integration will facilitate more efficient energy management and promote the development of renewable energy applications and electric mobility. The resultant advancements will contribute to the broader adoption of sustainable energy solutions and the enhancement of technological resilience in various industries.

### 4.3. Prospect

We conclude with a look at the future, highlighting areas for further exploration. First, the development of new HIs is essential. While the commonly used HIs, as mentioned above, can reflect the health state of the battery to a certain extent, their sensitivity and accuracy often fall short in complex scenarios. Therefore, future research should focus on developing composite HIs that integrate multiple physical and chemical parameters, such as temperature, pressure, and vibration, to enhance the comprehensiveness and accuracy of SOH assessments. Second, the emergence of new battery materials, such as solid electrolytes, high-nickel cathode materials, and silicon-based anode materials, presents new opportunities for improving battery performance. However, these materials also introduce new challenges for SOH evaluation. Thus, there is a need to deeply investigate the aging mechanisms of these new materials, including the interface stability of solid electrolytes and the degradation mechanisms of high-nickel cathode materials, to provide a robust theoretical foundation for SOH estimation. Additionally, the estimation of battery SOH involves multiple interdisciplinary areas, including battery technology, materials science, and data science. However, the collaboration between these disciplines is currently insufficient, which limits the depth and breadth of research. Therefore, we encourage researchers from various scientific fields to engage in joint research projects to foster the cross-fusion of knowledge and innovation. Finally, while machine learning and deep learning have made significant strides in the estimation of battery SOH, the interpretability and robustness of these models still require improvement. Future research should aim to develop highly interpretable machine learning models, enabling users to understand the decision-making processes of these models and enhancing their credibility. Combining these models with multi-task learning approaches can handle multiple tasks, such as SOH estimation, fault diagnosis, and life prediction, simultaneously, thereby improving the overall performance of the models. Through these efforts, more accurate and reliable estimates of battery SOH can be achieved, promoting the widespread application and sustainable development of battery technology.

## 5. Conclusions

Due to the increasingly widely used characteristics of lithium-ion batteries, the accurate estimation and prediction of SOH are becoming increasingly important, and clarifying the relationship between the aging mechanism and various HIs plays an essential role in improving the accuracy of the estimation model. Therefore, to make up for the gap in the discussion of the relationship between aging mechanism and HIs, the following discussion is carried out in this paper. First, the application of SOH estimation of lithium-ion batteries in energy storage and electric vehicles is introduced. Then, the mass use of HI is classified into capacity-related, resistance-related, temperature-related, and others. The model of extracting HIs to estimate the SOH is also briefly summarized. Finally, the development trend and challenges are discussed. With a representative HI to estimate the SOH, based on a non-destructive analysis of the battery, we can find the problem or abnormal situation inside the battery in time to avoid safety accidents caused by battery problems. At the same time, we can intuitively understand the use of the battery to develop a suitable charge and discharge strategy to extend its life. Through timely monitoring, the battery SOH reduces maintenance costs.

Linking the aging mechanism of lithium-ion batteries with the selected HI can improve the battery performance from the mechanism, extend the life, and achieve real-time monitoring. However, there are still some challenges in extracting HIs. Due to the complex mechanism of lithium aging, if we want to monitor SOH comprehensively, we need a large amount of data, which takes a long time and cannot meet the market demand for many retired batteries. In addition, there are still problems to be overcome in the quantitative analysis of LAM and LLI using HIs. Therefore, this review hopes to provide some reference for predicting lithium-ion batteries’ states and long-term development.

## Figures and Tables

**Table 1:** Summary of some existing review papers on estimating SOH of batteries.

Reference	Contribution	Drawback
[19]	Analysis of scientific and technical literature Advantages and disadvantages for online BMS Focus on accuracy and precision	Lack of characteristic quantity introduction No discussion on SOH and aging mechanism relationship
[20]	Review of SOH and RUL estimation models Classification and characterization identified Advantages and disadvantages in electric vehicle applications References for lithium-ion solar cell development.	Contribution to accurate SOH and RUL estimation Exploration of battery decay causes lacking Need for SOH–causes relationship analysis
[21]	Systematic review of cell modeling in BMS Coverage of commonly used state estimation methods Inclusion of all BMS states Discussion of various state estimation methods	Focus on BMS modeling and state estimation methods Coverage of all state estimation techniques Omission of battery aging mechanism in state estimation
[22]	Discussed the reason for the aging of LIBs Introduced the prediction methods based on classification frameworks Analyzed each methods main advantages and disadvantages	Only introduced SOH prediction of pure electric vehicles There is no extension of other new energy vehicle research There is no connection to the aging mechanism of lithium-ion batteries
[23]	Introduced the aging mechanism of LIBs Summarizing challenges and research prospects of SOH estimation Proposing new ideas for health prediction	Aging mechanism of both cathodes and anodes is summarized Summarizing the latest methods of SOH estimation Separating the link between the degradation mechanism and SOH
[24]	Internal and external aging mechanisms Influencing factors and their relationships Evaluation of capacity and power degradation	Complex, nonlinear whole-period variations need to be discussed

**Table 2:** Based on the IC/DV curve.

Authors	HI	Algorithm/Method	Advantage
[36]	IC peak Peak valley	Linear regression	Accurately estimate SOH and reduce computation time
[37]	IC peak value Corresponding voltage Energy Capacity determined by the IC peak voltage	SVR	Reduce the impact of noise and accurately predict SOH
[38]	IC peaks	Gaussian process regression	Accurately estimate SOH and reduce errors
[39]	IC peaks	SOH estimation method based on ICA	Accurately estimate SOH
[40]	IC peaks	GPR	Simulate real-life applications and reduce errors
[41]	IC multiple voltage intervals peaks	BPNN	Efficient capture of multiple voltage interval IC peaks
[42]	IC peaks	SVR	Data preprocessing for various scenarios
[43]	IC peaks	RVM	Good short-term forecasting and long-term forecasting stability
[44]	IC peaks Corresponding voltage	SVR	New data reconstruction methods and multi-view HIs improve the estimation accuracy
[45]	IC peak	logarithmic regression	The correlation between IC peaks and SOH changes was modeled to urate estimation capabilities at different magnifications
[46]	IC peak	SVR Multi-layer perceptron Random forest	Accurately estimate SOH and reduce errors
[47]	IC peak	Grey relational analysis Entropy weight method	Accurately estimate SOH and reduce errors
[48]	IC peak	SVR	It can be applied to battery modules and packs in different series–parallel configurations.
[49]	IC peaks and valleys	/	It proves the feasibility of ICA in electric vehicles.
[50]	IC peak height ratio	Linear regression Multiple linear regression	Prove SOH estimation from peak-to-height ratio
[51]	IC peak Valley Voltage	/	Verify suitability for battery capacity decay and SOH.
[52]	DRT quantization parameters	Neural network	Determine appropriate SOH indicators and achieve high-accuracy SOH estimation.
[53]	DV characteristic peaks	/	The gap between battery fatigue mechanism factors and impedance parameters is bridged
[55]	dV/dQ peak	/	Detecting the onset of lithium-plating
[56]	DV curve peak, valley	Coulomb counting method	Provide accurate online SOH calculations with low computational effort
[57]	The interval between two inflexion points in the DV curve	Linear regression	The estimation error is effectively reduced
[58]	The interval between two inflection points in the DV curve	Local data symmetry point	The resulting curve is smoother
[59]	Characteristics based on the DV curve	/	In the estimation of the SOH of the battery pack, it shows good performance
[60]	Characteristics based on the DV curve	KF PF	Estimated the SOC with a small error
[61]	Regional capacity in the DV curve	/	It shows the feasibility under different aging periods

**Table 6:** Form of ECM model.

Model	Circuit Structure	Voltage Expression	Disadvantage
Rint model	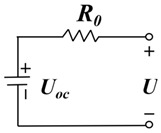	$U=U_{oc}-IR_{0}$	The application range is small without considering the charge transfer and diffusion polarization. When the current passing through the lithium-ion battery is significant, the deviation between the simulation results and the measured values of the model will be too large.
Thevenin model	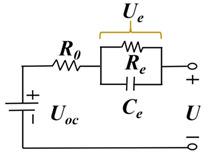	$U=U_{OC}-IR_{0}-U_{C}$ $U_{c}^{k}=U_{c}^{k-1}exp(-∆t/R_{e}C_{e})$	With the aging of the lithium-ion battery or when the temperature changes significantly, the internal impedance characteristics of the Li-ion battery will change from a single impedance arc to a double impedance arc or the capacity characteristics of a constant phase element, resulting in a decrease in the simulation accuracy of the model.
Second-order RC model	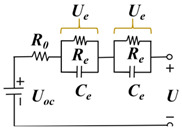	$U=U_{oc}-IR_{0}-U_{e}-U_{c}$ $U_{e}^{k}=IR_{e}\cdot \left[1-\exp\left(-\frac{∆t}{R_{e}C_{e}}\right)\right]+U_{e}^{k-1}exp(-∆t/R_{e}C_{e})$ $U_{c}^{k}=IR_{c}\cdot \left[1-\exp\left(-\frac{∆t}{R_{c}C_{c}}\right)\right]+U_{c}^{k-1}exp(-∆t/R_{c}C_{c})$	The frequency response range of this model is limited, and it cannot describe the behavior of some complex systems well. It is sensitive to the accuracy and stability of the parameters, and there will be seismic or unstable behavior.
PNVG model	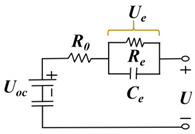	$U=U_{oc}-U_{Q}-U_{e}-IR_{0}$ $U_{Q}=I/C_{Q}$ $U_{e}=-\left(\frac{U_{e}}{R_{e}C_{e}}\right)+(I/C_{e})$	This model is computationally heavy and complex and does not apply to SOC estimation in practice.
GNL model	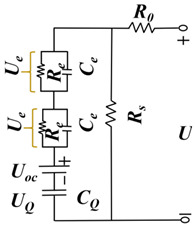	$U=\left[-\frac{R_{s}}{R_{s}+R_{e}}-\frac{R_{s}}{R_{s}+R_{e}}\right]\times [U_{1}U_{2}]-\frac{R_{s}R_{e}}{R_{s}+R_{e}}\cdot I+\frac{R_{s}}{\left(R_{s}+R_{e}\right)}\cdot U_{oc}$	The GNL model cannot capture the actual nonlinear behavior of the circuit when making simplified assumptions and only applies to other conditions; it has solid limitations and a lack of generality

## Data Availability

The original contributions presented in this study are included in the article/Supplementary Material. Further inquiries can be directed to the corresponding authors.

## Supplementary Materials

The following supporting information can be downloaded at: https://www.mdpi.com/1996-1944/18/1/145/s1, Table S1: a computational formula that converts a single feature into a statistical feature.

## Abbreviations

AI	Artificial intelligence	LLI	Loss of lithium ions
ANN	Artificial neural network	MAE	Mean absolute error
BMS	Battery management system	MKSVM	Multiple kernel relevance vector machine
BPNN	Bank-propagation neural network	ML	Machine learning
CE	Coulomb efficiency	MLECM	Mid and low-frequency domain ECM
CV	Cyclic voltammetry	MLR	Multiple linear regression
DTV	Differential thermal voltammetry	PDF	Probability density function
DTW	Dynamic time warping	PF	Particle filter
DVA	Differential voltage analysis	P2D	Pseudo-two-dimensional
ECM	Equivalent circuit model	QPSO	Quantum-behaved particle swarm optimization
EIS	Electrochemical impedance spectroscopy	RF	Random forest
EKF	Extended Kalman filter	RMSE	Root mean square error
ELM	Extreme learning machine	RUL	Remaining useful life
GPF	Genetic resampling particle filter	RVM	Relevance vector machine
GPR	Gaussian process regression	SEI	Solid electrolyte interface
HI	Health indictor	SOC	State of charge
ICA	Incremental capacity analysis	SOH	State of health
IMM	Interacting Multiple Model	SOP	State of power
ISVR	Incremental support vector regression	SVR	Support vector regression
KF	Kalman filter	WOA	Whale of a whale
LAM	Loss of active materials	ZSL	Zero-shot learning
LIB	Lithium-ion battery		
