# Supplementary material for: State of Health Estimation and Battery Management: A Review of Health Indicators, Models and Machine Learning
Source: Materials (Basel). 2025 Jan 2;18(1):145. doi: 10.3390/ma18010145 (PMC12068027; doi:10.3390/ma18010145)
Supplement: Supplementary file 1 [file materials-18-00145-s001.zip › materials-3348369-supplementary.pdf]

State of Health Estimation and Battery Management: A Review of Health Indicators, Models and Machine Learning

Mei Li [1], Wenting Xu [1], Shiwen Zhang [1], Lina Liu [1], Arif Hussain [1], Enlai Hu [1], Jing Zhang [1], Zhiyu Mao [2,3,*]
and Zhongwei Chen [2,3,*]

[1] College of Chemistry and Materials Science, Zhejiang Normal University, 688 Yingbin Avenue,
Jinhua 321004, China; liwangfanwen@zjnu.edu.cn (M.L.); Leonxu@zjnu.edu.cn (W.X.); 599232641@zjnu.edu.cn (S.Z.); linaliu@zjnu.edu.cn (L.L.); arifhussain@zjnu.edu.cn (A.H.); huenlai@zjnu.edu.cn (E.H.); jingzhang@zjnu.edu.cn (J.Z.)
[2] Power Battery & System Research Center, Dalian Institute of Chemical Physics, Chinese Academy of
Sciences, Dalian 116023, China
[3] State Key Laboratory of Catalysis, Dalian Institute of Chemical Physics, Chinese Academy of Sciences,
Dalian 116023, China
* Correspondence: zhymao@dicp.ac.cn (Z.M.); zwchen@dicp.ac.cn (Z.C.)

Supplementary material 1(S1)：Statistical characteristics of the calculation formula

Table S1: a computational formula that converts a single feature into a statistical feature

$Maxima(Max): x_{max}$
$Minima(Min):\ x_{min}$
$Mean: \bar{x} = \frac{1}{n}\sum_{i=1}^{n} x_i$
$Variance(Var):\ \mu_2 = \frac{1}{n-1}\sum_{i=1}^{n}(x_i - \bar{x})^2$
$Skewness(Ske): \mu_3 = \frac{1}{n}\sum_{i=1}^{n}(\frac{x-\bar{x}}{\sqrt{\mu_2}})^3$
$Excess\ Kurtosis(Kur):\ \mu_4 = \frac{1}{n}\sum_{i=1}^{n}(\frac{x-\bar{x}}{\sqrt{\mu_2}})^4 - 3$
